# Glycogen Repletion in Brown Adipose Tissue upon Refeeding Is Primarily Driven by Phosphorylation-Independent Mechanisms

**DOI:** 10.1371/journal.pone.0156148

**Published:** 2016-05-23

**Authors:** Christopher M. Carmean, Y. Hanna Huang, Matthew J. Brady

**Affiliations:** 1 From the Committee on Molecular Metabolism and Nutrition, University of Chicago, Chicago, Illinois, 60637, United States of America; 2 Department of Medicine, Section of Endocrinology, Diabetes and Metabolism, University of Chicago, Chicago, Illinois, 60637, United States of America; University of Minnesota - Twin Cities, UNITED STATES

## Abstract

Glycogen storage in brown adipose tissue (BAT) is generally thought to take place through passive, substrate-driven activation of glycogenesis rather than programmatic shifts favoring or opposing the storage and/or retention of glycogen. This perception exists despite a growing body of evidence suggesting that BAT glycogen storage is actively regulated by covalent modification of key glycogen-metabolic enzymes, protein turnover, and endocrine hormone signaling. Members of one such class of covalent-modification regulators, glycogen-binding Phosphoprotein Phosphatase-1 (PP1)-regulatory subunits (PPP1Rs), targeting PP1 to glycogen-metabolic enzymes, were dynamically regulated in response to 24 hr of starvation and/or 24 hr of starvation followed by *ad libitum* refeeding. Over-expression of the PPP1R Protein Targeting to Glycogen (PTG), under the control of the aP2 promoter in mice, inactivated glycogen phosphorylase (GP) and enhanced basal- and starvation-state glycogen storage. Total interscapular BAT glycogen synthase and the constitutive activity of GS were conditionally affected. During starvation, glucose-6-phosphate (G-6-P) levels and the relative phosphorylation of Akt (p-Ser-473-Akt) were both increased in PTG-overexpressing (Tg) mice, suggesting that elevated glycogen storage during starvation modifies broader cellular metabolic pathways. During refeeding, Tg and WT mice reaccumulated glycogen similarly despite altered GS and GP activities. All observations during refeeding suggest that the phosphorylation states of GS and GP are not physiologically rate-controlling, despite there being a clear balance of endogenous kinase- and phosphatase activities. The studies presented here reveal IBAT glycogen storage to be a tightly-regulated process at all levels, with potential effects on nutrient sensing *in vivo*.

## Introduction

BAT is a multi-depot thermogenic organ, responsive to changes in ambient temperature or nutritional status. Though BAT has historically been recognized for its role in thermal homeostasis during hibernation [1], the medical community has recently taken an interest in the proliferation and activation of BAT in humans to expend excess calories, restoring the balance between caloric intake and expenditure [2, 3]. Thermogenesis in BAT depends upon a regulated intracellular program of elevated glucose and lipid uptakes from the blood stream, rapid oxidation of fatty acids, and the expenditure of the stored chemical potential energy from these substrates as heat [4]. While these mechanisms have been well studied over the past several decades, far less attention has been given to the similarly-dynamic metabolic process of glycogen storage in BAT.

The observation that interscapular BAT (IBAT) in mice possesses high concentrations of glycogen, second only to those of the liver under basal conditions [5, 6], has received relatively little attention. When perturbed, IBAT glycogen has been observed in mice at concentrations of 0.02–40 mg glycogen/g tissue, representing both an impressive upper limit of accumulation as well as a dynamic 2000-fold physiological range [5, 6]. There are two interventions through which IBAT glycogen has been observed to span this large range; 1) in the case of starvation or prolonged partial starvation followed by refeeding, where energy deficit depletes glycogen and recovery during refeeding induces marked over-accumulation [5, 7, 8], and 2) changes to housing temperature, where acclimation to the cold depletes glycogen and a return to warmth induces over-accumulation [6, 9]. These phenomena have been sporadically reported over the past 80 years without clarification of the molecular mechanism(s) by which BAT glycogen storage is regulated [1, 10].

Glycogen particle expansion and degradation are enzymatically regulated by glycogen synthase (GS) and glycogen phosphorylase (GP), respectively. GS catalyzes the addition of uridine diphosphate-glucose (UDP-glucose) onto the terminal glucosyl moiety of glycogen chains, a reaction that is subject to regulation at the levels of substrate availability (modulated via glucose transport), total GS protein levels, spatial localization of GS to glycogen particles, and constitutive GS activity [11–13]. GS can be activated by allosteric binding of the first substrate formed upon entry of glucose into the glycolytic pathway, glucose-6-phosphate (G-6-P), and progressively inhibited by phosphorylation at any of 9 target serine sites [14, 15]. Allosteric binding of G-6-P is capable of maximally activating GS, regardless of phosphorylation state [11, 16, 17]. GP has a single serine that serves as its sole phosphorylation site, which increases its constitutive activity. In this way, GS and GP are inversely regulated by phosphorylation events, where GS and GP are inhibited and activated, respectively, by phosphorylation.

Dephosphorylation events targeting regulatory serines on GS and GP are carried out by protein phosphatase-1 (PP1). In glycogen metabolism, scaffolding proteins known as glycogen-targeting PP1 regulatory subunits (PPP1Rs) co-localize the catalytic subunit of PP1 (PP1c) with specific glycogen-metabolic enzymes [18–24], fine-tuning glycogen flux [22, 24].

The PTG gene (called PPP1R3C in humans), coding for the peptide PTG, is expressed in all tissues that have been examined except the testis [19]. PTG possesses 3 major domains; a glycogen-binding domain, a PP1-binding domain, and a common binding site for GS or GP [25]. PTG over-expression in 3T3-L1 adipocytes increases glycogenic activity by targeting PP1 phosphatase activity against both GS and GP [26, 27]. Knockdown or dominant-negative mutant expression experiments collectively suggest that GP is the preferred target of endogenous PTG [28, 29]. Despite initial reports of homozygous lethality in a PTG global-knockout, [30] there have been two separate reports that PTG global-knockout mice are protected from high-fat diet-induced hepatic steatosis, glucose intolerance, and peripheral insulin resistance [31]. These mice had reduced skeletal muscle mTORC1/SREBP activity, suggesting that that low glycogen stores may decrease lipogenesis and that high glycogen levels may activate it [31]. Such findings highlight the complexities of glycogen regulation and function.

Despite the wide range of glycogen storage observed in IBAT, little is known about the mechanisms by which GS and GP are controlled in this tissue. In the current work, the relative contributions of covalent vs. allosteric regulation of these enzymes under three physiological conditions were investigated: *ad libitum* fed, 24 hr of starvation, and 1–2 hr of refeeding following starvation. Evidence is presented that glycogen storage in the *ad libitum* fed or starved state is modulated by the phosphorylation state of GP, regulated by glycogen-targeted PP1. Conversely, during refeeding, glycogen replenishment proceeds primarily through the allosteric activation of GS that overrides the regulatory phosphorylation status of the enzyme. Data also suggests that glycogen itself may serve as an intracellular metabolic rheostat, feeding back into broader metabolic control pathways via alterations in glucose-6-phosphate levels and the phosphorylation of Akt.

## Materials and Methods

### Transgenic Mice

CD1 mice possessing an additional copy of the endogenous gene for Protein Targeting to Glycogen (PTG), under the control of the aP2 promoter (detailed in [32]), were back-crossed for 8 generations onto the C57BL/6J background and then maintained on that background for the duration of the described studies. All “Tg” labels refer to mice heterozygous for the aP2-PTG transgene.

### Animal Handling, sacrifice, and tissue collections

Mice were maintained on a 12:12-hr light:dark cycle (lights on at 6:00 AM) under specific pathogen-free conditions in the Gwen and Jules Knapp Center for Biomedical Discovery animal facility at the University of Chicago at 22°C. Experimental interactions with the mice were performed under dim red lighting during the dark phase to minimize circadian disruption. Only male mice were used for all experiments to eliminate potential sex-specific variation. All animal procedures for this study were approved by the University of Chicago Institutional Animal Care and Use Committee, and all efforts were made to minimize animal suffering. For sacrifice, mice were anaesthetized with Isoflurane vapor (Baxter Inc., NDC 10019-773-60) and death was ensured by cervical dislocation. IBAT was immediately harvested and placed in liquid nitrogen.

### Feeding, Starvation, and Refeeding

Male, group-housed, wild-type (WT) or Tg littermates were either sacrificed at 19:30 (Fed), or starved for 24 (S) beginning at 19:30 and then either sacrificed or allowed to refeed *ad libitum* for 1, 2, or 4 hr (SR 1, SR 2, or SR 4, respectively) prior to sacrifice. The cages of S and SR mice were changed at the time of food withdrawal. Except during starvation, all mice were provided *ad libitum* access to irradiated Harlan Teklad Global 18% Protein Rodent Diet (Harlan Laboratories, #1918), referred to as chow and kept on corn-cob bedding.

### Glycogen Determination

Glycogen was measured in IBAT exactly as described previously [5].

### Western Blotting

Whole individual IBAT pads were homogenized by sonication (Sonics VibraCell Ultrasonic Processor) in 15 uL homogenization buffer (50 mM Hepes pH 7.4, 150 mM NaCl, 10 mM NaF, 1 mM EDTA, 5% v/v glycerol, 0.5% v/v Triton X-100) containing 1% v/v protease inhibitor cocktail (Sigma P8340) per mg tissue. Samples were centrifuged at 4°C for 15 minutes at 13,400xg to separate lipids and insoluble components. Infranatants were transferred to fresh tubes and centrifuged at 4°C for 15 minutes at 13,400xg. Protein concentration was determined by BCA assay (Pierce BCA Protein Assay Kit Prod #23227). Homogenate drawn from the post-spin infranatant was mixed with Laemmli buffer at a volumetric ratio of 3:1 homogenate:Laemmli and heated to 100°C for 10 minutes. Each well of an SDS-polyacrylamide gel was loaded with protein homogenate containing equal amounts of total protein in Laemmli buffer or 2–5 uL of protein ladder (Cat # 161–0374, Bio-Rad). Separation took place at room temperature by electrophoresis at 120V for 1–2 hr. Proteins were then transferred onto polyvinylidene fluoride membranes (0.2 μm pore size, Cat #162–0177, Bio-Rad) in a wet transfer apparatus at 20V for 14–18 hours at room temperature and then immunoblotted with either rabbit anti-pan-GP (generated and purified as described in [33]) or rabbit anti-phospho-GP (diluted 1:1000), generated and purified as described in [33]). Mouse anti-pan-Akt primary antibody (diluted 1:2000, #2920, Cell Signaling) was used as a loading control. To blot phosphorylated Akt at Ser473, a rabbit anti-phospho-Akt (diluted 1:5000, #4060S, Cell Signaling) antibody was used, with total Akt (as described above) utilized as a loading control.

### Quantitative RT-PCR

For each biological replicate, one whole snap-frozen IBAT pad was pulverized while frozen and mRNA was isolated using the DNeasy blood and tissue spin column kit (Qiagen). cDNA was prepared from 500 ng of mRNA using the qScript cDNA synthesis DNA kit. Perfecta SYBR Green Fastmix (Quanta Biosciences) was used to perform quantitative real-time PCR (MyIQ iCycler real-time PCR thermal cycler, Bio-Rad). See Table 1 for primer sequences. For analysis, the comparative delta Ct method was used as described previously [34]. The Ct value for the reference transcript was subtracted from the Ct value of the target transcript, providing a delta Ct value. The fold-change between target and reference was determined using the following equation: Fold Change_ref_ = 2^-(delta Ct)^. This manipulation normalized the expression values, allowing us to scale the data with accurate error bars and perform statistical comparisons between conditions. To calculate fold-change between conditions and genotypes, each Fold Change_ref_ value was divided by the mean Fold Change_ref_ of the control condition to obtain a measure of relative expression.

**Table 1:** Quantitative, real-time PCR primer sequences.

Gene	Forward Primer (5’-3’)	Reverse Primer (5’-3’)
**Acetyl-CoA Carboxylase**	TACTGCCATCCCATGTGC	GCTTCCAGGAGCAGTCGT
**ATP Citrate Lyase**	AGCGATCCGAAGAGTTGG	GTTCTTTGCCGGTCTGCT
**β-Actin**	AGAGGGAAATCGTGCGTGAC	CAATAGTGATGACCTGGCCGT
**ChREBPα**	CGACACTCACCCACCTCTTC	TTGTTCAGCCGGATCTTGTC
**Fatty Acid Synthase**	GGCTCTATGGATTACCCAAGC	CCAGTGTTCGTTCCTCGGA
**GYS1**	GGCTGAGAGGCATGGCTACTG	ACTCTACCGGTCACCAAGTCT
**Hexokinase 1**	TATCGGTCCAGCACGTATGC	AGAACCGTCTACGCCAACTG
**Hexokinase 2**	TGATCGCCTGCTTATTCACGG	AACCGCCTAGAAATCTCCAGA
**Insulin Receptor**	TTCAGGAAGACCTTCGAGGATTACCTG	AGGCCAGAGATGACAAGTGACTCCTT
**IRS1**	GCCAGAGGATCGTCAATAGC	GAGGAAGACGTGAGGTCCTG
**IRS2**	CGGCGAATGTTCATAAGCTGC	AACCTGAAACCTAAGGGACTGG
**Leptin**	TGTCCAAGATGGACCAGACTC	ACTGGTCTGAGGCAGGGAGCA
**PPP1R3B**	TGGTTCAGACAGGGACACAT	GGCAAACTCCATTCTTTCGT
**PPP1R3C total**	CCTTCCAGAAGAACCAGC	CTCAGTTGGAATGACACG
**PPP1R3C endogenous**	ATGCCCGTGGACATGGCCAT	GTGTGGGCTCTTCCACTCACT
**PPP1R3D**	GCTGAAGGTACCGAGGACAT	TAGTCGCGACCATCATTGTT
**PYGM**	CTTAGCCGGAGTGGAAAATGT	GTAATCTCTCGGAGTAGCCACA
**GLUT1**	CAGTTCGGCTATAACACTGGTG	GCCCCCGACAGAGAAGATG
**GLUT4**	ACTCTTGCCACACAGGCTCT	AATGGAGACTGATGCGCTCT
**Uncoupling Protein 1**	ACTGCCACACCTCCAGTCATT	CTTTGCCTCACTCAGGATTGG

### G-6-P assay

One whole IBAT pad from each mouse was homogenized by sonication in 15 volumes of ice-cold PBS and placed on ice. Homogenates were centrifuged at 16,100xg for 3 minutes at 4°C and the infranatant was transferred to 10 kDa spin columns (Cat #431478, Corning). Samples were centrifuged at 16,100xg for approximately 20 minutes at 4°C. The concentration of G-6-P in the filtrate was determined using a commercially-available high-sensitivity G-6-P assay kit (Cat #ab107923, Abcam).

### Statistical analysis

Each comparison made between two treatments or conditions was performed with a Mann-Whitney rank sum test. Significance was set at *p<0*.*05*.

## Results

### aP2-PTG mice over-express PTG in IBAT

**Fig 1:**
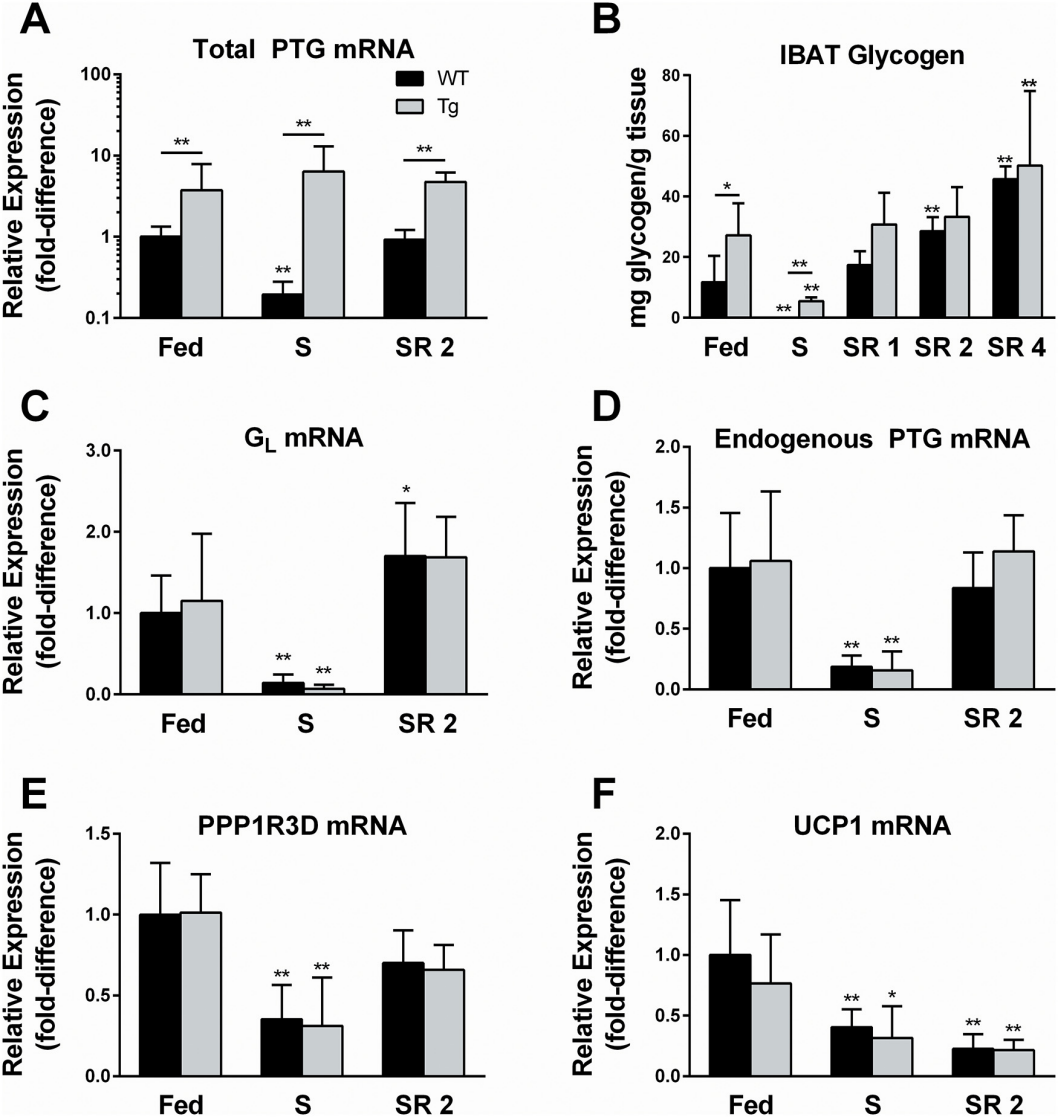
Effects of Protein Targeting to Glycogen over-expression on interscapular brown adipose tissue (IBAT) glycogen. IBAT was excised and snap-frozen from 8-week old mice either *ad libitum* chow-fed (Fed), 24-hr starved (S), or starved for 24 hr then allowed to refeed for 1, 2, or 4 hr (SR 1, SR 2, SR 4, respectively) before sacrifice. IBAT glycogen (6 mice per condition and time point) (A), and relative gene expressions for genes of interest (5 mice per condition and time point) (B-F) were determined. Error bars are ±SD. Statistics: A Mann-Whitney Rank Sum test was performed between genotypes within each condition (significance indicated above each line indicating the comparison) as well as between the Fed state and each other condition within each genotype (stars directly over the bar). For gene expressions, comparisons were made between Fed and S conditions for each transcript *p<0.05, **p<0.01.

IBAT has the second highest basal concentration of glycogen in the mouse, second only to that of the liver [5], making the nature of its regulation and its physiological significance an important question in BAT physiology. PTG was previously shown to regulate glycogen storage in multiple tissues [19, 28, 32, 35–37] and was endogenously expressed in IBAT. An existing transgenic (Tg) mouse line in which PTG was over-expressed under the control of the aP2 promoter [32], exhibited elevated total PTG mRNA expression in IBAT under all conditions and time points measured (Fig 1A).

### PTG over-expression in adipose tissues increased IBAT glycogen

IBAT glycogen levels were measured in wild-type and Tg mice fed *ad libitum* (Fed) and in mice starved for 24 hr and then refed for 0, 1, 2, or 4 hr followed by sacrifice and tissue harvest (S, SR 1, SR 2, or SR 4, respectively). Over-expression of PTG in IBAT significantly increased basal glycogen storage (Fig 1B). Starvation significantly lowered IBAT glycogen levels in both genotypes, however Tg mice partially preserved IBAT glycogen storage during starvation. After 1 hr of refeeding, IBAT glycogen content returned to fed-state levels for each genotype. Though PTG expression in the IBAT of Tg mice was significantly elevated during refeeding, the concentration of IBAT glycogen was not significantly different between genotypes during the three refeeding time points measured (Fig 1B).

### PPP1Rs are expressed in IBAT and dynamically regulated

The expression levels of PPP1Rs were previously shown to have profound effects on glycogen storage in muscle, WAT, and liver, and to change dramatically in response to physiological stimuli. Therefore, it was of interest whether these subunits would be differentially-expressed in response to starvation, refeeding, or altered glycogen-metabolic status brought about by the over-expression of PTG. Three of the seven known glycogen-targeting PP1-regulatory subunits (G_L_, PTG, and PPP1R3D) were robustly expressed and dynamically-regulated in both WT and Tg IBAT, decreasing in response to starvation and rebounding during refeeding (Fig 1C–1E). G_L_, expression significantly increased by 70% during refeeding over the Fed state (Fig 1C) in WT IBAT. Gene expression of the muscle-specific glycogen-targeting regulatory subunit, G_M_, was barely-detectable under all conditions examined, and completely undetectable in multiple samples (data not shown). UCP1 expression decreased in response to starvation and remained suppressed after 2 hr of refeeding with no genotype-specific differences observed (Fig 1F).

Endogenous PTG expression in all conditions measured was not affected by expression of exogenous PTG (Fig 1D). The significant increase in PPP1R3B expression between WT Fed and refed mice was lost in Tg mice (Fig 1C). No significant genotype-specific differences in expression were observed in 24 hr-starved mice for the following genes: acetyl-coA carboxylase, ATP citrate lyase, carbohydrate-response element binding protein α, fatty acid synthase, hexokinase 1, hexokinase 2, Insulin receptor, insulin receptor substrate 1, insulin receptor substrate 2, leptin, solute carrier 2 member 1, solute carrier 2 member 4 (data not shown).

### Measurement of G-6-P levels and Akt phosphorylation

**Fig 2:**
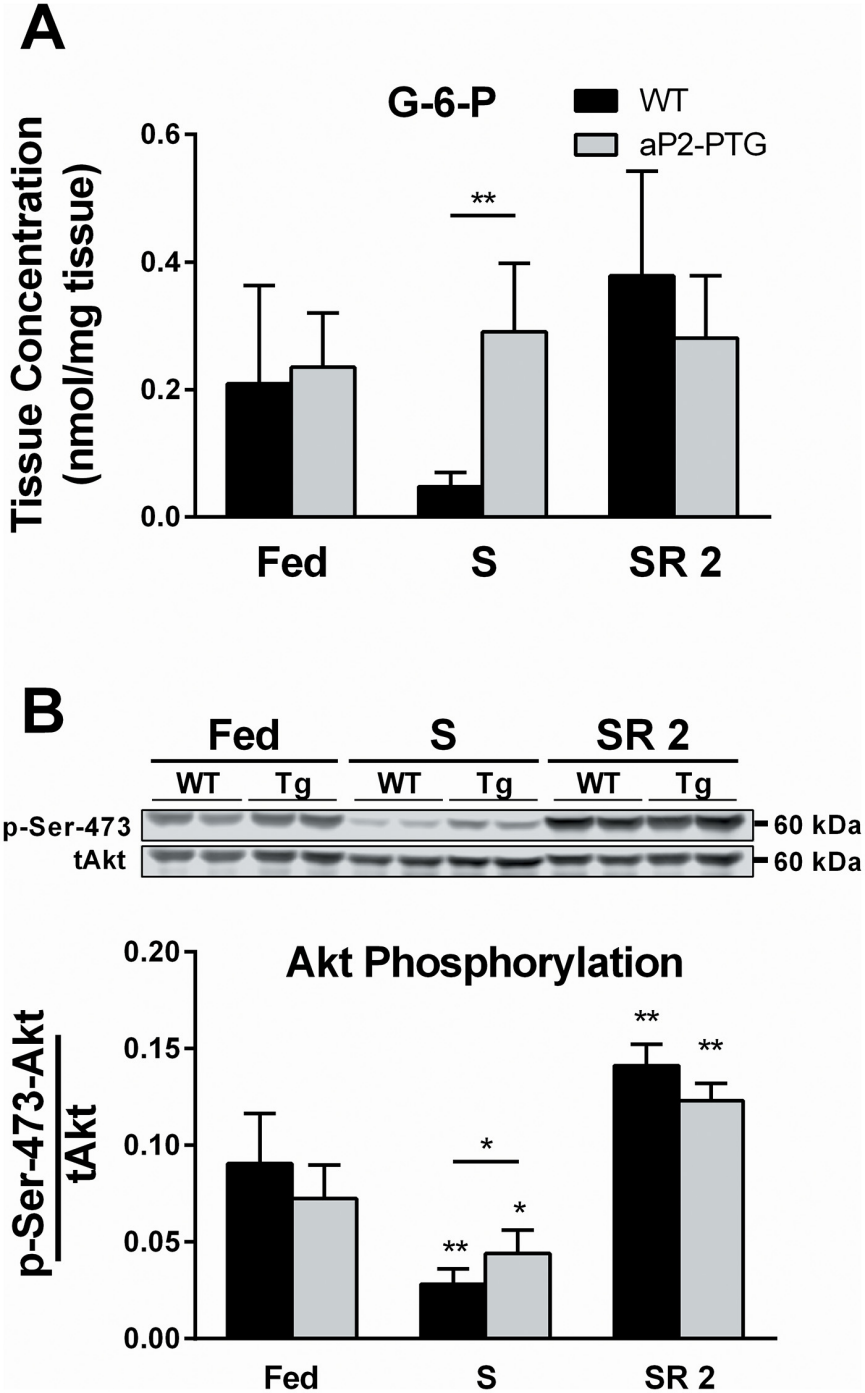
Effects of PTG over-expression on glucose-6-phosphate (G-6-P) and phosphorylation of Akt in starved mice. Intracellular G-6-P (A) and p-Ser-473-Akt protein (B) was determined in excised, snap-frozen IBAT from WT and Tg male 5–7 week-old mice sacrificed in the Fed, S, or SR 2 states as described in “Experimental Procedures.” N = 5 mice for G-6-P in each condition and genotype. N = 6 for p-Ser-473-Akt in each condition and genotype spread across 3 western blots (2 biological replicates from each condition, time point, and genotype per blot, 30 ug protein loaded per well as determined by BCA assay). Error bars are ±SD. Statistics: A Mann-Whitney rank sum test was performed within each genotype against the Fed state (stars directly over individual bars), as well as between genotypes in each condition (stars over lines indicating comparisons). *p<0.05, **p<0.01.

To evaluate the potential contribution of G-6-P as a driver of glycogen synthesis, its concentration was measured in the IBAT of Fed, S, or SR 2 mice (Fig 2A). After 24 hr of starvation, G-6-P was 6-fold higher in Tg IBAT compared to WT littermates. During refeeding, G-6-P levels were similar to Fed-state levels and not significantly different between genotypes. To examine whether the starvation-state increase in G-6-P in Tg mice was a result of increased glucose uptake, the phosphorylation of Akt at Ser473 (p-Ser-473-Akt), an activator of glucose uptake, was measured by western blot (Fig 2B). Tg mice exhibited elevated p-Ser-473-Akt levels in the starved state, indicating increased Akt activity. Though increased Akt phosphorylation and G-6-P may be expected to indicate increased GLUT4 translocation to the plasma membrane, preliminary experiments suggested that there were no differences in total or plasma membrane-associated GLUT4 between Tg and WT mice (data not shown).

### Constitutive GS activity is selectively altered in Tg mice

**Fig 3:**
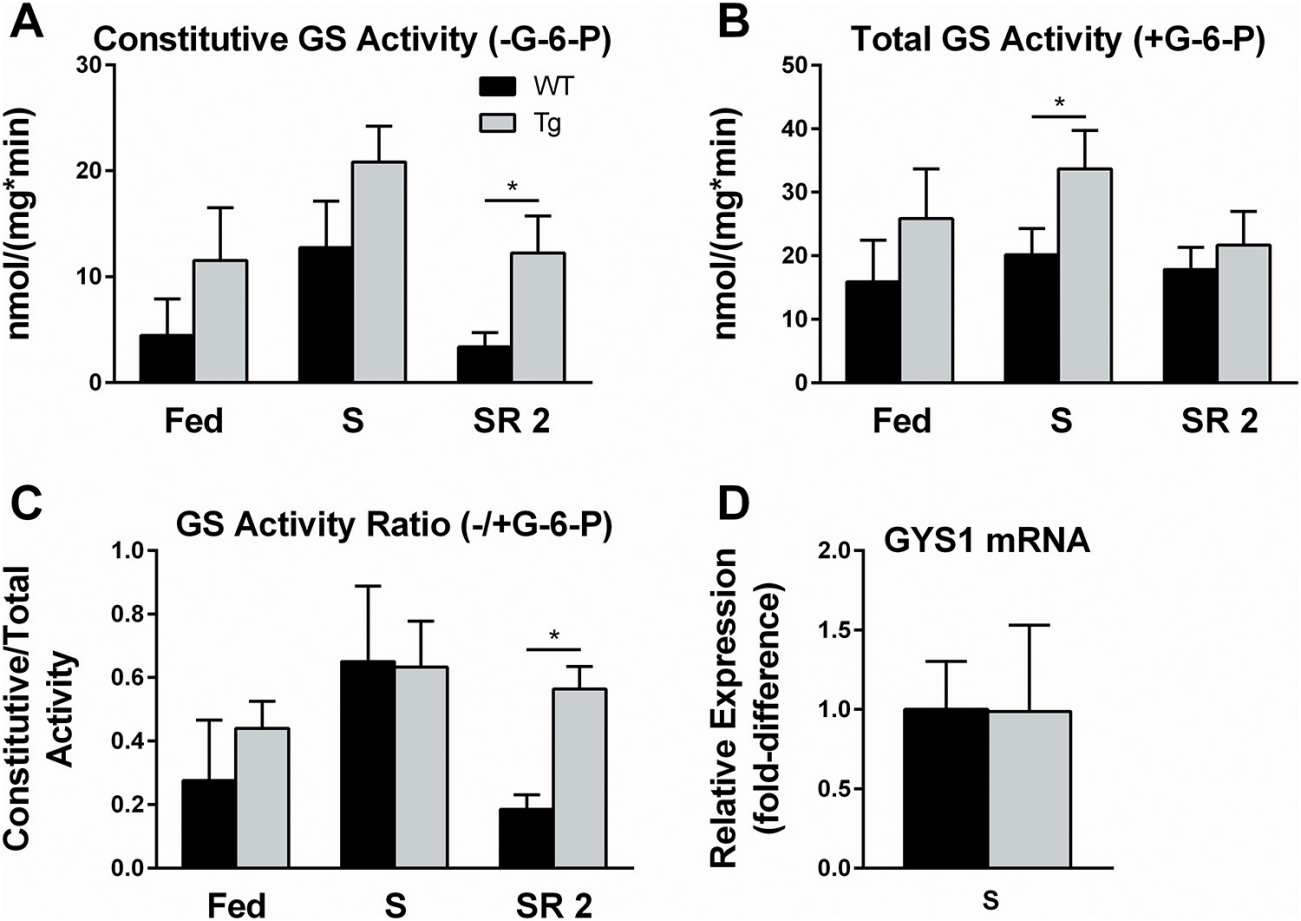
Effects of nutritional perturbations and PTG over-expression on GS. IBAT from Fed, S, or, SR 2 WT and Tg 5–7 week-old mice was excised and snap frozen as described in “Experimental Procedures.” IBAT was homogenized and the ability of glycogen synthase in the homogenate to convert UDP-glucose to glycogen was determined in the absence *(A)* or presence *(B)* of G-6-P. The ratio of these activities *(C)* was then calculated. N = 5 for each condition and genotype. Error bars are ±SD. Statistics: A Mann-Whitney rank sum test was performed within each genotype against that of the Fed state (stars directly over individual bars), as well as between genotypes in each condition (stars over lines indicating comparisons). *p<0.05, **p<0.01.

It was unknown whether IBAT glycogen storage was primarily controlled allosterically (by substrate availability) or actively regulated (by covalent modification) of key glycogen-metabolic enzymes such as GS and GP. To determine whether GS was actively regulated by nutritional status, GS activities (Fig 3A–3C) were measured in Fed, S, or SR 2 mice. Because constitutive GS activity is increased by dephosphorylation and decreased by phosphorylation, the relative constitutive activity of GS between two conditions can be thought of as a comparison between the net kinase and phosphatase pressures exerted on the enzyme. Total GS activity, which is measured in the presence of saturating quantities of its allosteric activator G-6-P, is considered a measure of total GS protein in the sample.

Total GS activity was 66% higher in starved Tg mice over starved WT littermates but not significantly different under other conditions examined (Fig 3B). Constitutive GS activity was significantly greater in Tg mice over WT littermates after 2 hr of refeeding (Fig 3A), indicating reduced phosphorylation of GS. To compare the regulatory state of GS, the constitutive activity of GS was normalized to total GS activity (Fig 3C). Refeeding for 2 hr significantly increased both the constitutive GS activity as well as the ratio of constitutive:total GS activity in Tg mice relative to their WT refed littermates, indicating that PTG over-expression altered the regulation of GS phosphorylation in the refed state, but not in the basal or starved states. GYS1 mRNA was compared between the starved states as a potential explanation for the observed increase in total GS, but was not different between genotypes (Fig 3D).

### PTG mediates the dephosphorylation of GP

**Fig 4:**
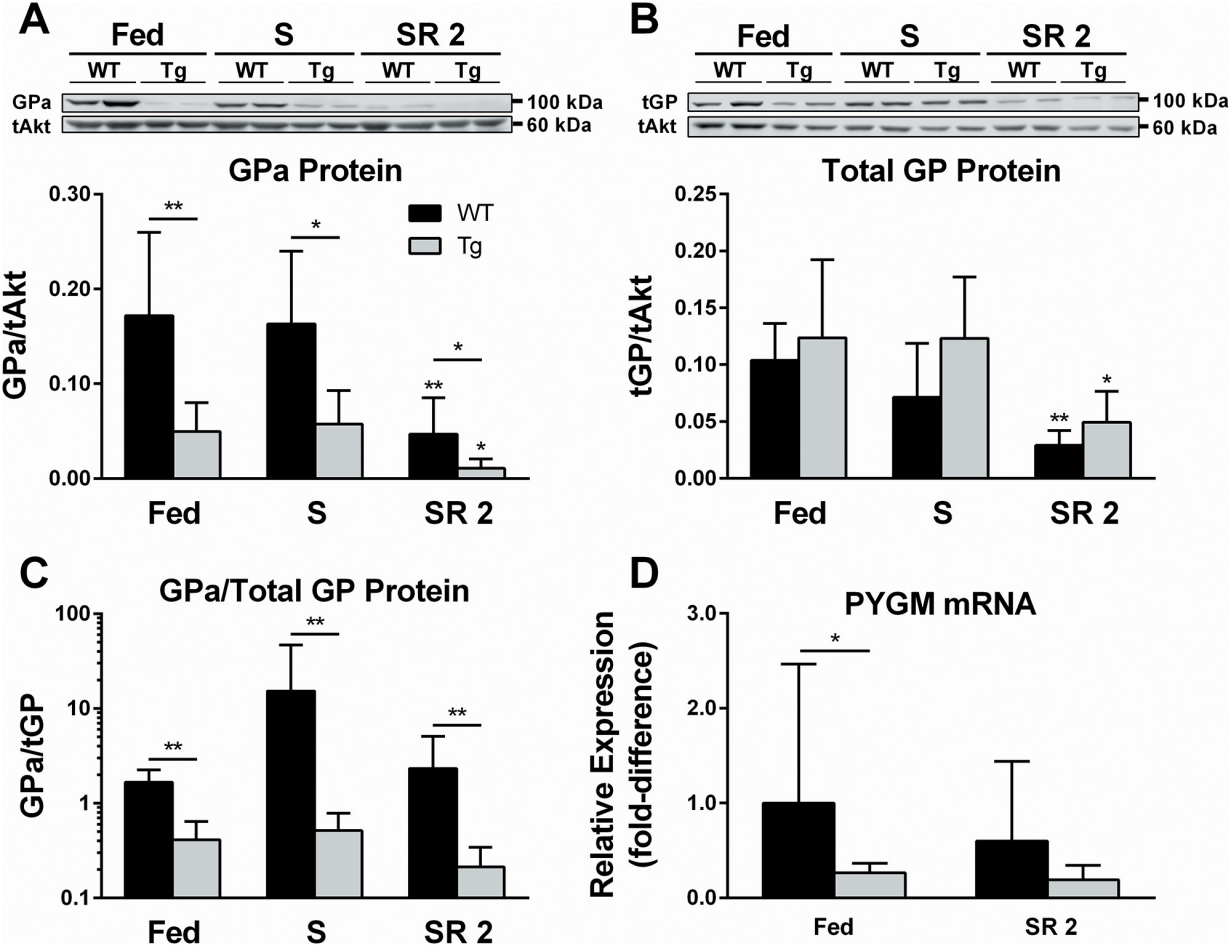
Effects of nutritional perturbations and PTG over-expression on GP. Phospho-glycogen phosphorylase (GP_a_) *(A)* and total glycogen phosphorylase (tGP) *(B)* protein levels were measured in snap-frozen IBAT excised from 5–7 week-old WT and Tg mice sacrificed in the Fed, S, or SR 2 states as described in “Experimental Procedures.” N = 6 per condition and genotype, spread out across 3 western blots (2 biological replicated from each condition, time point, and genotype per blot, 30 ug protein loaded per well as determined by BCA assay). Relative signal was quantified by densitometry and normalized to total Akt signal. Representative blots are shown above each densitometry chart. Error bars are ±SD. Statistics: A Mann-Whitney rank sum test was performed within each genotype against the Fed state (stars directly over individual bars), as well as between genotypes in each condition (stars over lines indicating comparisons) *p<0.05, **p<0.01.

The GP-catalyzed breakdown of glycogen into G-1-P is a rate-limiting step in glycogenolysis. Despite this fact, GP is commonly overlooked as a major player in the regulation of glycogen storage in insulin-sensitive, glycogenic tissues such as muscle [11, 13]. The few reports in the literature in which GP activity was biochemically determined during thermal or nutritional challenges in IBAT conflict each other, leaving the questions completely open of whether GP protein levels and/or phosphorylation state would be modified in the context of nutritional stress or PTG over-expression in IBAT. Over-expression of PTG consistently decreased the phosphorylation state of IBAT GP protein relative to WT littermates in all conditions measured (Fig 4A) without causing any genotype-specific changes in total GP (Fig 4B). This observation was confirmed by examining the ratio of phosphorylated GP to total GP protein (Fig 4C), demonstrating a decrease in phosphorylated GP after adjusting for total GP protein in all conditions and time points measured. During refeeding, both the total- and phosphorylated GP protein levels were decreased in both genotypes (Fig 4A and 4B), which would be expected to reduce glycogen breakdown. Changes in PYGM mRNA expression did not correlate with changes in GP protein, as mRNA expression was not significantly different between the Fed and SR 2 states for each respective genotype (Fig 4D).

## Discussion

There were several results in this study that challenge the commonly-held view of BAT glycogen storage from that of a passive, overflow pathway, supporting instead a model of multi-layered, active regulation, in which both substrate-, covalent-, and expression-level mechanisms govern glycogen storage. Firstly, our finding that G_L_, PPP1R3D, and PTG were all robustly expressed and dynamically regulated in IBAT, while the skeletal muscle glycogen-targeting PP1-regulatory subunit, G_M_, was poorly expressed or undetectable was surprising. IBAT shares a precursor cell population with skeletal myocytes, which utilize G_M_ for catecholamine-sensitive promotion of glycogen synthesis. Despite this fact, G_M_, at least at the expression level, was not consistently expressed or dynamically regulated in IBAT. The combination of PPP1R expressions observed in IBAT more closely mirrored that of the liver, where G_L_, PPP1R3D, and PTG are the primary (though not the only) actors in the regulation of glycogen metabolism [22, 38]. This implies that the phospho-regulatory pressures exerted on GS and GP in IBAT are distinct from those in skeletal muscle or white adipose tissue. Because the GS-activating effects of G_L_ are allosterically inhibited by GP*a* (which is not the case for G_M_), the phosphorylation state of GS in IBAT may be more dependent upon the regulation of GP than in skeletal muscle. In IBAT, where catecholamines induce thermogenesis (and would in parallel be expected to regulate phosphorylase kinase activity) this allows for both GS and GP to be coordinated at the level of targeted PP1-mediated dephosphorylation, enabling both enzymes to be up- or downregulated simultaneously.

Though PTG over-expression reduced the phosphorylation of GP in all conditions examined, its effects on GS were conditional. Total GS in the starved state was elevated, which was likely an indirect effect of PTG over-expression. PTG has been shown in cell culture to increase GS localization to glycogen particles, which stabilizes GS, reducing its turnover [39]. Because glycogen levels were significantly higher in the IBAT of starved Tg mice than their WT littermates, exogenous PTG may have facilitated an increase in GS stability through enhanced glycogen storage, which would be anticipated to have a feed-forward effect on glycogen storage. Despite awareness of these possibilities, the primary mechanism by which starvation-state glycogen was increased in Tg mice remains indeterminable from the presented data. The combination of increased relative phosphorylation of Akt, increased substrate availability (G-6-P levels), and reduced GP activity, could all have contributed materially to the observed increase in glycogen storage. Differences in circulating insulin levels could have also contributed to the results obtained, a metric that was not measured due to past observations of normal insulinemia in fasted Tg mice. During refeeding, glycogen storage proceeded similarly in both genotypes, despite increased constitutive GS activity and decreased constitutive GP activity. That the marked elevation of constitutive GS activity and the decrease in GPa levels had no effect on glycogen storage relative to WT littermates during refeeding strongly suggests the storage of glycogen during refeeding is not dependent upon the physiological ranges of phosphorylation states of these two glycogen-metabolic enzymes. This is supported by the Brady laboratory’s previous work, which showed that, when hormonal parameters known to affect glucose uptake were manipulated *in vivo*, changes in glycogen storage were at least partly driven by glucose uptake and intracellular substrate availability [5]. It is not possible to definitively tease out whether G-6-P levels were allosterically stimulating glycogen synthase activity well beyond the constitutive activity conferred by its phosphorylation state, or whether some combination of substrate availability, spatial regulation, and/or alternative allosteric regulatory mechanisms were limiting synthesis such that altering the phosphorylation states of GS and GP was inconsequential. Rather, we can confidently say that changes to the phosphorylation states of GS and GP favoring glycogen storage did not have an effect during refeeding.

That constitutive GS activity was elevated during refeeding in Tg mice is in agreement with prior reports in which the ability of PTG overexpression to activate GS was demonstrated [26, 28, 32]. The observed dependence upon nutritional state might result from the refeeding-associated decrease in total GP, potentially reducing the amount of GP associated with PTG, thereby allowing more GS to bind PTG and be dephosphorylated. This would suggest that GP is the preferred binding target for PTG, but that in the absence of GP, PTG possesses sufficient affinity for GS to bind it and direct PP1 activity against it. This explanation would align with prior published reports in which both GS and GP were shown to be targeted by PTG, yet the dephosphorylation of GP predominated [28]. Alternatively, the reduction in GP*a*, combined with the reduction in total GP, may have alleviated GP*a*-mediated repression of G_L_. This would have allowed G_L_ to exert greater PP1-targeting effects towards GS, producing the observed phenotype. Further study is required to delineate these potential mechanisms.

Our collective observations support the hypothesis that the phosphorylation states of GS and GP are regulated properties *in vivo*, but that kinase and phosphatase activities balance each other out, creating a stable tension between activating and deactivating forces. It appears that the primary mechanisms by which glycogen is variably regulated *in vivo* during refeeding are independent of changes to the phosphorylation states of GS and GP, leaving substrate availability, localization effects, and decreased glycogenolysis as the prime suspects. It is therefore plausible to hypothesize that IBAT is constantly primed to synthesize glycogen at all times, and relying on substrate availability and the rate of glycogenolysis to govern the net concentration of glycogen.

In Tg mice, the relative phosphorylation of Akt was unaffected by elevated glycogen levels under basal conditions (Fed), but the starved state yielded multiple indicators of altered signal transduction and glucose metabolism. The enhanced phosphorylation of Akt in the starved state, combined with the elevation of G-6-P, suggests alteration to a major nutrient response signaling pathway. If this is the case, then it challenges the prevailing notion that glycogen acts simply as an overflow pathway for glucose storage during times of excess glucose uptake; that instead, it plays a regulatory role in Akt phosphorylation and potentially broader metabolic pathways such as lipogenesis, lipolysis, glucose uptake, or thermogenesis.

Glycogen levels during starvation may feed back on endocrine and nutrient-sensing pathways via Akt. Ser-473 on Akt is a target of nutrient-sensing mTORC2 kinase activity [40]. When phosphorylated, Akt enhances glucose uptake and allows for the provision of additional substrate to pathways such as glycogenesis, oxidative phosphorylation, and glycolysis. It also reduces inhibition of mTORC1 by phosphorylation and inactivation of TSC1, an inhibitor of mTORC1 [41]. The recent observation in a PTG knockout model suggesting that aberrantly-low glycogen levels may inhibit mTORC1 activity, ultimately causing a reduction in hepatic lipogenesis [31], provides a plausible link between PTG expression and metabolic homeostasis. The previously published model suggested that low glycogen suppresses mTORC1, while our results provide evidence that increasing starvation-state glycogen levels positively regulates elements upstream of the mTORC1 pathway.

The described studies, taken in the context of past reports, suggest that BAT possesses a covalently-regulated glycogen storage program controlled by changes in GP activity, acting as a feed-forward metabolic regulator by altering Akt phosphorylation during starvation and rapidly over-accumulating during recovery from starvation independently of the phosphorylation states of GS and GP.
